# Associations between complex multimorbidity, activities of daily living and mortality among older Norwegians. A prospective cohort study: the HUNT Study, Norway

**DOI:** 10.1186/s12877-020-1425-3

**Published:** 2020-01-21

**Authors:** Siri H. Storeng, Kristin H. Vinjerui, Erik R. Sund, Steinar Krokstad

**Affiliations:** 1 Department of Public Health and Nursing, Faculty of Medicine and Health Sciences, Norwegian University of Science and Technology, NTNU, Trondheim, Norway; 2 HUNT Research Centre, Department of Public Health and Nursing, Faculty of Medicine and Health Sciences, Norwegian University of Science and Technology, NTNU, Levanger, Norway; 3 Faculty of Nursing and Health Sciences, Nord University, Levanger, Norway; 4 Levanger Hospital, Nord-Trøndelag Hospital Trust, Levanger, Norway

**Keywords:** Activities of Daily Living, Aged, Complex Multimorbidity, HUNT Study, Norway

## Abstract

**Background:**

With increasing age, having multiple chronic conditions is the norm. It is of importance to study how co-existence of diseases affects functioning and mortality among older persons. Complex multimorbidity may be defined as three or more conditions affecting at least three different organ systems. The aim of this study was to investigate how complex multimorbidity affects activities of daily living and mortality amongst older Norwegians.

**Methods:**

Participants were 60–69-year-olds at baseline in the Nord-Trøndelag Health Study 1995-1997 (HUNT2) *n* = 9058. Multinomial logistic regression models were used to investigate the association between complex multimorbidity in HUNT2, basic and instrumental activities of daily living in HUNT3 (2006–2008) and mortality during follow-up (*n* = 5819/5836). Risk ratios (RR) and risk differences (RD) in percentage points (pp) with 95% confidence intervals (CI) were reported.

**Results:**

47.8% of 60–69-year-olds met the criteria of complex multimorbidity at baseline (HUNT2). Having complex multimorbidity was strongly associated with the need for assistance in IADL in HUNT3 11 years later (RR = 1.80 (1.58–2.04) and RD = 8.7 (6.8–10.5) pp) and moderately associated with mortality during the follow-up time (RR = 1.22 (1.12–1.33) and RD = 5.1 (2.9–7.3) pp). Complex multimorbidity was to a lesser extent associated with basic activities of daily living 11 years later (RR = 1.24 (0.85–1.83) and RD = 0.4 (− 0.3–1.1) pp).

**Conclusions:**

This is the first study to show an association between complex multimorbidity and activities of daily living. Complex multimorbidity should receive more attention in order to prevent future disability amongst older persons.

## Background

The world is experiencing population aging where the number of people over 60 years is expected to more than double and to be over 2 billion in 40 years [1]. Health- and long-term care increases with age, as do health care costs [1]. In 2015 23% of the burden of disease occurred in people 60 years and older [2] and age-related diseases account for 51% of the years of life lost and lived with disability [3]. More than half of the older population has co-occurrence of several chronic diseases [4]. Multimorbidity is commonly defined as the coexistence of two or more chronic conditions requiring long-term care [5–7]. However, increasing the cut-off to three or more conditions increases specificity and differentiation among older persons [8]. Further, complex multimorbidity defined as three or more chronic conditions affecting three or more different body systems is suggested to better identify patients needing care from different specialists than multimorbidity [8]. Multimorbidity has in systematic reviews been found to be associated with functional decline [9], poor quality of life [10] and increased mortality [11] amongst older persons. There has been found gender and socioeconomic differences where women and lower educated groups have higher prevalence of multimorbidity than men and higher educated groups [12–16]. Both country specific and common global disease combination patterns have been found [17–20]. But it is of greater importance to study the disability associated with the conditions rather than counting diseases and comparing patterns [6] and there is a need for more research to determine the consequences of multimorbidity [21].

Disability may be defined as the “*gap between personal capability and environmental demand”* [22] and measured by the need for assistance in activities of daily living [23]. Activities of daily living can be divided into basic (ADL) and instrumental activities of daily living (IADL) [24, 25]. Basic activities include abilities necessary for fundamental functions such as eating and walking, whereas instrumental activities concern functions required for living in a community such as shopping and taking the bus. A large systematic review from 2015 including 37 studies concluded that multimorbidity predicts future functional decline [9]. However, the majority were cross-sectional studies, only six studies included activities of daily living as outcome. Complex multimorbidity has been suggested to better identify high-need individuals [8] and to our knowledge this will be the first study that investigates the associations between complex multimorbidity, activities of daily living and mortality.

### Aim

The aim of this study was to investigate the association between complex multimorbidity, basic and instrumental activities of daily living and mortality among older participants of the Nord-Trøndelag Health Study, HUNT2 (1995–97) and HUNT3 (2006–2008) in a prospective cohort study.

## Methods

### Material

**Fig. 1:**
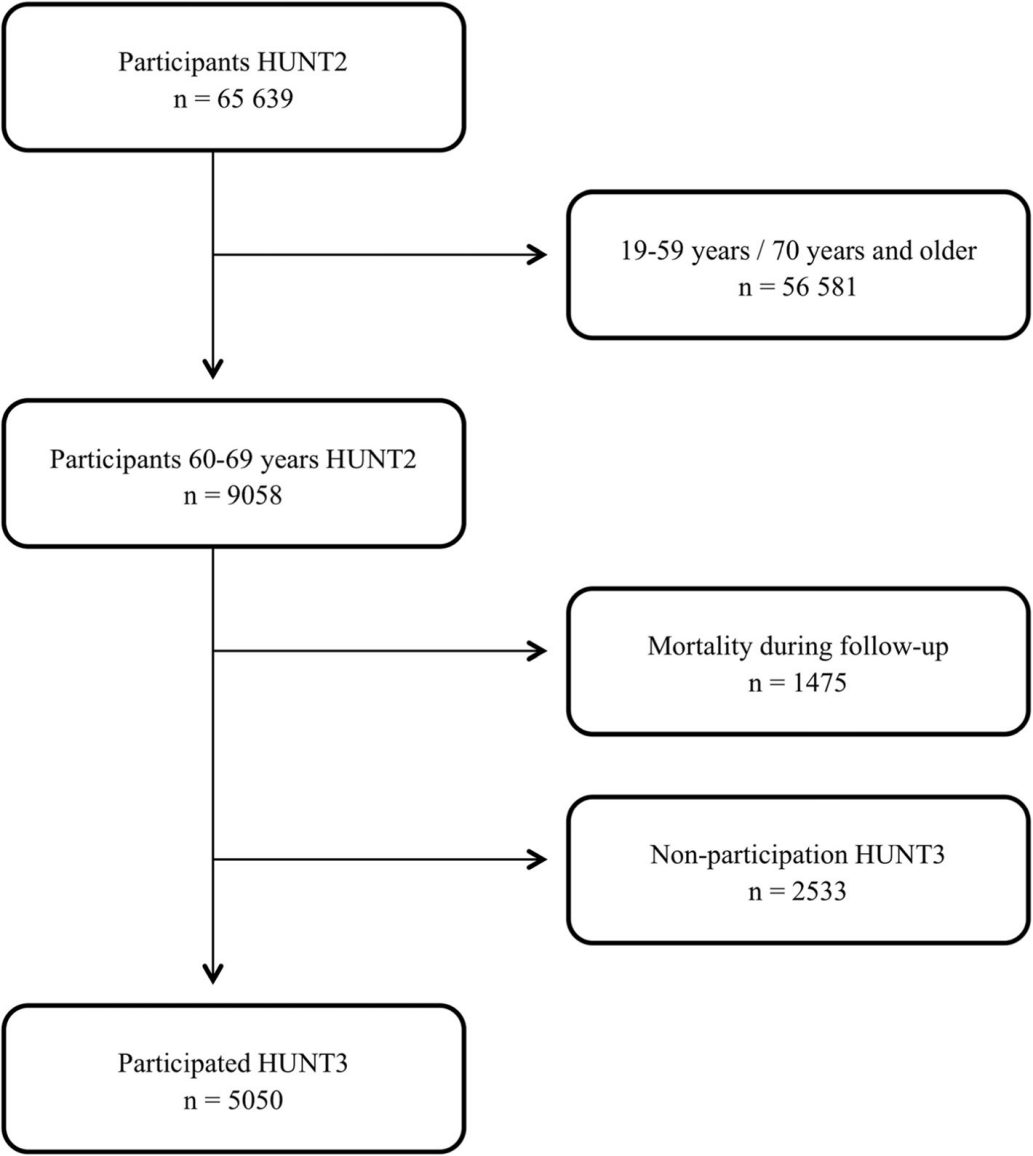
Flow chart showing selection of participants

The HUNT Study is a population-based health study where all participants aged 20 years and older in the county of Nord-Trøndelag were invited to participate. There have been four waves of data collection comprising HUNT1 (1984–96), HUNT2 (1995–97), HUNT3 (2006–08) and a fourth wave completed in 2019 (HUNT4, 2017–19). The participants filled out questionnaires and undertook a clinical screening test. All participants signed a written consent to participate and the Regional Ethical Committee approved the HUNT Study [26] as well as this project. The HUNT Study is extensively described elsewhere [26]. The material in this prospective cohort study was 60–69-year-olds participating in HUNT2 at baseline. A flow chart of the participants included in this study is shown in Fig. 1. Of the 9058 60–69-year-olds in HUNT2 5050 also participated in HUNT3, 1475 died during the follow-up time (1995–2008) and 2533 did not participate in HUNT3. The overall participation rate was 69.5 and 54.1% in HUNT2 and HUNT3 respectively, but the participation rate in the middle-aged was higher (85.6% in 60–69-year-olds in HUNT2 and 71.1% in HUNT3) [26, 27].

### Variables

The main predictor of interest in this study was complex multimorbidity measured at baseline in HUNT2 (1995–97). Complex multimorbidity was measured by compiling a complete list of 38 conditions in HUNT2 (Additional file 1), as this has been found to identify individuals with a high need of care [8]. The conditions were classified according to their chapter in the 10th revision of the International Classification of Diseases (ICD-10) and complex multimorbidity was defined as having three conditions from three different organ systems. Lastly, the variable was dichotomized into fulfilling the criteria for complex multimorbidity or not. Conditions included in the complex multimorbidity variable were self-reported or measurements (blood pressure, cholesterol level and obesity) and cut-offs were defined according to available validated criteria. Question texts, answer categories, operationalization, ICD-10 classification and studies on the validity of the self-reported conditions included in the complex multimorbidity variable is provided in Additional file 1.

The outcomes in this study were defined as needing assistance from another person in one or more basic or instrumental activities of daily living in HUNT3 (2006–08). Basic activities of daily living included to walk, go to the toilet, wash oneself, shower, dress, go to bed and get up, and eat. Instrumental activities of daily living included preparation of meals, light and heavy housework, do laundry, go shopping, take the bus, take medicines, and go out. Since population health change can be regarded as a continuum of disease, disability, loss of function and mortality [28], ADL and IADL disability was compared to the competing outcome mortality during follow-up from HUNT2 to HUNT3. Confounders included in the statistical models were the socio-demographic variables age (continuous), sex (men/women) and education (primary, secondary, tertiary). Question texts, answer categories and handling of confounders and outcome variables are provided in Additional files 2 and 3.

### Statistical calculations

Multinomial logistic regression models were used to investigate the association between complex multimorbidity at baseline in HUNT2 (1995–97) and basic and instrumental activities of daily living in HUNT3 (2006–08) and mortality during follow-up. In a sensitivity analysis non-participation in HUNT3 was included as a competing outcome in the multinomial logistic regression analysis to evaluate its effect on the results. The analyses were adjusted for relevant confounders (age, sex and education). The postestimation command *adjrr* in Stata was performed to attain risk ratios (RR) and risk differences (RD) with corresponding 95% confidence intervals (95% CI) [29]. All analyses were performed in Stata version 15 [30].

## Results

Table 1 shows that the prevalence of complex multimorbidity amongst 60–69-year-olds in HUNT2 was 47.8% (*n* = 4327). 91.4% (*n* = 8277) of the participants had primary and secondary education. The prevalence of complex multimorbidity varied by education; 52.1% with primary education fulfilled the criteria for complex multimorbidity compared to 35.5% with tertiary education. There was a 11.9 percentage points gender difference in prevalence of complex multimorbidity between women and men.

**Table 1:** Prevalence and sociodemographic characteristics of complex multimorbidity in HUNT2 (1995–1997), *n* = 9058

	No CMM (%)	CMM (%)
Age		
60–64	2334 (53.2)	2053 (46.8)
65–69	2397 (51.3)	2274 (48.7)
Total	4731 (52.2)	4327 (47.8)
Missing	0	0
Education		
Primary	2134 (47.9)	2324 (52.1)
Secondary	2097 (54.9)	1722 (45.1)
Tertiary	488 (64.6)	268 (35.5)
Total	4719 (52.2)	4314 (47.8)
Missing	12 (48.0)	13 (52.0)
Sex		
Women	2193 (46.5)	2520 (53.5)
Men	2538 (58.4)	1807 (41.6)
Total	4731 (52.2)	4327 (47.8)
Missing	0	0

*Abbreviations* used in the table: *CMM* Complex Multimorbidity, *HUNT* The Nord-Trøndelag Health Study

Among the 60–69-year-olds in HUNT2 2.4% reported needing assistance from another person in any of the basic activities of daily living in HUNT3, whereas 19.9% needed assistance in instrumental activities of daily living (Table 2). Doing heavier housework, doing laundry and taking the bus were the activities where most participants reported needing assistance from another person, with 13.1, 8.2 and 7.2%, respectively. Descriptive statistics of exposure and confounders in HUNT2 by outcome categories in HUNT3 is shown in Additional file 4.

**Table 2:** Prevalence of ADL and IADL disability in HUNT3 (2006–2008), *n* = 5050

ADL disability		IADL disability	
Walk	43 (1.0)	Warm meals	141 (3.3)
Toilet	32 (0.7)	Light housework	76 (1.8)
Wash yourself	50 (1.2)	Heavy housework	564 (13.1)
Bath/shower	82 (1.9)	Laundry	347 (8.2)
Dress	40 (0.9)	Pay bills	168 (3.9)
Go to bed and get up	34 (0.8)	Take medicines	56 (1.3)
Eat	22 (0.5)	Go out	69 (1.6)
		Do the shopping	142 (3.3)
		Take the bus	301 (7.2)
No ADL	4270 (97.6)	No IADL	3488 (80.1)
Any ADL	104 (2.4)	Any IADL	869 (19.9)
Sum ADL	4374 (86.6)	Sum IADL	4357 (86.3)
Missing	676 (13.4)	Missing	693 (13.7)
Total	5050 (100)	Total	5050 (100)

*Abbreviations* used in the table: *ADL* Basic Activities of Daily Living, *HUNT* The Nord-Trøndelag Health Study, *IADL* Instrumental Activities of Daily Living

Table 3 shows that those with complex multimorbidity were on average 24 (− 15–83) % more likely to have ADL disabilities compared with those without complex multimorbidity, with an absolute risk difference of 0.4 (− 0.3–1.1) percentage points. Having complex multimorbidity increased the risk for mortality during follow-up from HUNT2 to HUNT3 on average with 22 (12-33) % or 5.1 (3.0–7.3) percentage points. The risk ratios did not change when including non-participants in HUNT3 as a competing outcome in the multinomial logistic regression analysis, but the risk difference decreased slightly (0.1 and 1.4 percentage points for ADL disability and mortality respectively, see Additional file 5).

**Table 3:** Association between complex multimorbidity (HUNT2) and ADL (HUNT3), multinomial logistic regression,^a^ *n* = 5836

CMM	ADL independent		ADL disability		Mortality during follow-up	
	RR (95% CI)	RD (95% CI)	RR (95% CI)	RD (95% CI)	RR (95% CI)	RD (95% CI)
No	1.0 (ref)	0.0 (ref)	1.0 (ref)	0.0 (ref)	1.0 (ref)	0.0 (ref)
Yes	0.93 (0.90–0.96)	−5.5 (−7.7–3.3)	1.24 (0.85–1.83)	0.4 (−0.3–1.1)	1.22 (1.12–1.33)	5.1 (3.0–7.3)

*Abbreviations* used in the table: *ADL* Basic Activities of Daily Living, *CI* Confidence Interval, *CMM* Complex Multimorbidity, *HUNT* The Nord-Trøndelag Health Study, *RD* Risk Difference, *ref*. Reference category, *RR* Risk Ratio

^a^Adjusted for age, sex and education

Table 4 shows that those with complex multimorbidity were on average 80 (58–104) % more likely to have IADL disabilities compared to those without complex multimorbidity, with an absolute risk difference of 8.7 (6.8–10.5) percentage points. Having complex multimorbidity increased the risk for mortality during follow-up from HUNT2 to HUNT3 with 22 (12–33) %, with an absolute risk difference of 5.1 (2.9–7.3) percentage points. The risk ratios were not altered by including non-participants in HUNT3 as a competing outcome in the multinomial logistic regression analysis, but the absolute risk differences decreased with 2.7 and 1.5 percentage points for IADL disability and mortality respectively (Additional file 6). There was an interaction between sex and IADL disability where men with complex multimorbidity had lower risk of IADL disability (ratio of relative risks = 0.68 (0.49–0.93)). However, the effect estimates did not change after including the interaction term, and the main effects models is presented in Table 4.

**Table 4:** Association between complex multimorbidity (HUNT2) and IADL (HUNT3), multinomial logistic regression,^a^ *n* = 5819

CMM	IADL independent		IADL disability		Mortality during follow-up	
	RR (95% CI)	RD (95% CI)	RR (95% CI)	RD (95% CI)	RR (95% CI)	RD (95% CI)
No	1.0 (ref)	0.0 (ref)	1.0 (ref)	0.0 (ref)	1.0 (ref)	0.0 (ref)
Yes	0.79 (0.76–0.83)	−13.8 (− 16.2 - -11.4)	1.80 (1.58–2.04)	8.7 (6.8–10.5)	1.22 (1.12–1.33)	5.1 (2.9–7.3)

*Abbreviations* used in the table: *CI* Confidence Interval, *CMM* Complex Multimorbidity, *HUNT* The Nord-Trøndelag Health Study, *IADL* Instrumental Activities of Daily Living, *RD* Risk Difference, *ref*. Reference category, *RR* Risk Ratio

^a^Adjusted for age, sex and education

## Discussion

In this prospective cohort study 47.8% of 60–69-year-olds met the criteria of complex multimorbidity at baseline in HUNT2 (1995–97). Having complex multimorbidity was strongly associated with the need for assistance in IADL in HUNT3 11 years later (2006–08) and moderately associated with mortality during the follow-up time.

Few comparable studies have reported prevalence of complex multimorbidity, even though it is proposed to better identify patients in high need of care than multimorbidity [8]. In an Australian study 17.0% of the population was found to have complex multimorbidity [31], but no age limits or age-specific prevalence estimates were reported. We found a 60.5% prevalence of multimorbidity amongst 60–69-year-olds using a cut-off of 3 out of 38 conditions. This in line with prevalence estimates from a systematic review studying multimorbidity in older persons [32]. In HUNT2 it has previously been found a 62% prevalence of multimorbidity (defined as 2 or more out of 21 conditions) for participants aged 60 years [33]. The inclusion of more conditions in this study (38 conditions) could explain the similar prevalence despite different cut-offs.

This is to our knowledge the first study showing to what degree complex multimorbidity increases the future risk for disability in instrumental activities of daily living, adding to previous research on multimorbidity and disability. A systematic review from 2015 concluded that multimorbidity predicts future functional decline in adults, but comparisons between studies are hampered by the heterogeneity in definitions and operationalizations of multimorbidity and functional decline and the included cohort studies had short follow-up time (1–3 years) [9]. Later studies including four cross-sectional and two cohort studies with 1 and 2 years follow-up time have also found associations between multimorbidity and ADL/IADL disability [34–38]. It may seem that disease combinations including depression and cognitive impairment increase the risk for ADL/IADL disability substantially compared to combinations of only somatic disease [37, 39]. Other studies have found associations between the number of chronic conditions and function in both basic and instrumental activities of daily living [18, 40, 41]. In a cross-sectional study including 567 participants 80 years and older multimorbidity was found to be associated with disability [42] but in a cohort study with 3 years follow-up time including the same participants multimorbidity predicted mortality and hospitalization but not functional decline [43]. A Chinese study including 52,667 participants over 80 years found that the association between multimorbidity and ADL disabilities became stronger between 1998 and 2008 [44]. Thus, the associations between multimorbidity and function may change over time and be different among the oldest old.

The declining association between complex multimorbidity and IADL disability, ADL disability and mortality during follow-up, could indicate a hierarchical relationship between instrumental and basic activities of daily living and mortality [45, 46], and these could be seen as successive stages in population health changes [28, 47]. Differing comorbidity patterns have been found to be differentially associated with functional ability [48–51]. Co- and multimorbidity-patters in HUNT2 have already been studied [33] but their association with function should be investigated in a future study. There was a weaker association between men with complex multimorbidity and IADL disability compared with women with complex multimorbidity. This is in line with a previous meta-analysis and systematic reviews that have found women to have higher prevalence and to be more strongly associated with multimorbidity compared with men [12, 15, 16]. The results from this study can be generalized to community-dwelling older populations comparable to the Norwegian setting with low mortality and a high number of older persons. It cannot be generalized to institutionalized older persons, since very few of them have been included in the HUNT Study.

The main limitations of this study are healthy survivor and participant bias and the lack of information about activities of daily living at baseline. Institutionalized older persons and those not able to attend the HUNT Study are not included. Non-participants in HUNT3 have been found to have higher mortality, lower socioeconomic status and higher prevalence of several chronic diseases but also lower prevalence of some conditions [52]. Further, the participation rate for people aged 60–69 years decreased from 85.6% in HUNT2 to 71.1% in HUNT3 [26, 27]. Healthy participant and survivor bias were evaluated in a sensitivity analysis including non-participation in HUNT3 and mortality during follow-up as competing outcomes to ADL/IADL disability in a multinomial logistic regression model. This did not affect the relative risks but decreased the absolute risk differences slightly. Thus, the associations between complex multimorbidity and ADL disability, IADL disability and mortality could be slightly overestimated due to non-participation bias.

Since we did not have information about activities of daily living in HUNT2 (questions were only asked to participants older than 70 years) we were unable to control for this at baseline. Abilities to perform basic activities such as walking and eating are fundamental for independent living and may also be determinants for participation in the HUNT Study. Therefore, the HUNT data may represent the healthier part of the older adult population. If participants with ADL/IADL disabilities were included at baseline this would have introduced differential misclassification bias where those with ADL/IADL disabilities at baseline were more likely to be classified as ADL/IADL disabled in HUNT3 compared with those who were ADL/IADL independent in HUNT2. This could have led to both over- and underestimation of the results [53]. Despite using a longitudinal study design with on average 11 years follow-up time, the lack of control for ADL/IADL status at baseline could introduce reverse causality and thereby explain some of the associations. A recent narrative literature proposes a synergistic effect of multimorbidity and functional decline on health, quality of life and survival, and that there could be a bidirectional relationship between multimorbidity and function with common underlying pathways [54].

The HUNT2 questionnaire did not include common conditions such as chronic obstructive pulmonary disease, alcohol misuse, health failure and only one gastro-esophageal and respiratory disorder (gastro-esophageal reflux disease and asthma). However, defining complex multimorbidity from a complete list of the available conditions, as we did in HUNT2, should identify individuals with a high need of care [8]. The validity of individual self-reported conditions has been found to be varying (Additional file 1), but most people with multimorbidity should be identified by using self-report [55]. Lastly, there were few participants reporting ADL disabilities and large uncertainties associated with small absolute effect estimates. However, this is a group that is likely to need a high level of care and the indication that having complex multimorbidity is associated with ADL disability 11 years later is an interesting finding.

## Conclusion

A high prevalence of complex multimorbidity was found in this Norwegian population with older persons and this is the first study to show to what degree complex multimorbidity is associated with instrumental activities of daily living. This could indicate that the load of having several diseases itself is important and should receive attention in addition to treatment of the individual conditions. Focusing on complex multimorbidity could be instrumental in order to prevent future functional decline amongst older persons.

## Supplementary information

**Additional file 1.** Variables, question texts, answer categories and operationalization of conditions included in complex multimorbidity variable (HUNT2).

**Additional file 2.** Question texts, answer categories and operationalization of confounders (HUNT2).

**Additional file 3.** Question texts, answer categories and operationalization of outcome variables in HUNT3.

**Additional file 4.** Exposures and confounders in HUNT2 (1995–97) by outcomes in HUNT3 (2006–08).

**Additional file 5 **Association between complex multimorbidity (HUNT2) and ADL (HUNT3), mortality and non-participation (HUNT3), multinomial logistic regression.* *n* = 8357.

**Additional file 6 **Association between complex multimorbidity (HUNT2) and IADL (HUNT3), mortality and non-participation (HUNT3), multinomial logistic regression.* *n* = 8340.

## Data Availability

The Nord-Trøndelag Health Study (HUNT) has invited persons aged 13–100 years to three surveys between 1994 and 2008 and is now running a new survey (HUNT4) since 2017. Comprehensive data from more than 125,000 persons having participated at least once and biological material from 78,000 persons are collected. The data are stored in HUNT databank and biological material in HUNT biobank. HUNT Research Centre has permission from the Norwegian Data Inspectorate to store and handle these data. The key identification in the data base is the personal identification number given to all Norwegians at birth or immigration, whilst de-identified data are sent to researchers upon approval of a research protocol by the Regional Ethical Committee and HUNT Research Centre. To protect participants’ privacy, HUNT Research Centre aims to limit storage of data outside HUNT databank and cannot deposit data in open repositories. HUNT databank has precise information on all data exported to different projects and are able to reproduce these on request. There are no restrictions regarding data export given approval of applications to HUNT Research Centre. For more information see: http://www.ntnu.edu/hunt/data.

## Abbreviations

ADL: Activities of Daily Living
CI: Confidence Interval
CMM: Complex Multimorbidity
HUNT: The Nord-Trøndelag Health Study
IADL: Instrumental Activities of Daily Living
pp: Percentage points
RD: Risk Difference
RR: Risk Ratio
