# Supplementary material for: Associations between complex multimorbidity, activities of daily living and mortality among older Norwegians. A prospective cohort study: the HUNT Study, Norway
Source: BMC Geriatr. 2020 Jan 21;20:21. doi: 10.1186/s12877-020-1425-3 (PMC6974981; doi:10.1186/s12877-020-1425-3)
Supplement: Supplementary file 1 — Additional file 1. Variables, question texts, answer categories and operationalization of conditions included in complex multimorbidity variable (HUNT2). [file 12877_2020_1425_MOESM1_ESM.docx]

## Additional File 1. Variables (validity), question texts, answer categories and operationalization of conditions included in complex multimorbidity variable (HUNT2).

| **Variable (HUNT2)** | **Question text** | **Answer categories** | **Operationalization** | **Missing** | **Type** | **ICD-10 chapter** |
| --- | --- | --- | --- | --- | --- | --- |
| Cancer (1) | Do you have or have you ever had cancer? | No  Yes | Yes/no | Missing = "no" if answered any of the other yes/no questions in the same block [epilepsy, cancer, mental disease, other disease] | Diagnosis | II Neoplasms |
| Hypothyroidism (2) | Have you ever had hyperthyroidism (too high metabolism)? | No  Yes | Yes/no | Missing = "no" if answered any of the other yes/no questions in the same block [hyperthyroidism, hypothyroidism, goiter, other disease in the thyroid] | Diagnosis | IV Endocrine / nutritional / metabolic |
| Hyperthyroidism (2) | Have you ever had hypothyroidism (too low metabolism)? | No  Yes | Yes/no | Missing = "no" if answered any of the other yes/no questions in the same block [hyperthyroidism, hypothyroidism, goiter, other disease in the thyroid] | Diagnosis | IV Endocrine / nutritional / metabolic |
| Diabetes (3) | Have you had or do you have diabetes? | No  Yes | Yes/no | Missing = "no" if answered any of the other yes/no questions in the same block [MI, angina, stroke, diabetes] | Diagnosis | IV Endocrine / nutritional / metabolic |
| Obesity (4) | Manual measurement of height and weight with the participants wearing light clothes without shoes. | Height in whole cm  Weight in kilos, rounded to the nearest half kilo | BMI > 30 *or* BMI 25-29 + waist circumference ≥ 88 cm (women) or 102 cm (men) | Measurements, not corrected for missing | Risk factor | IV Endocrine / nutritional / metabolic |
| Dyslipidemia (5) | Measurement performed in non-fasting serum from fresh blood samples. Measured by applying an enzymatic colorimetric cholesterol esterase method in mmol/l. | Mmol/L | Hypercholesterolemia ≥ 8.0 mmol/L | Measurement, not corrected for missing. | Risk factor | IV Endocrine / nutritional / metabolic |
| Insomnia (6) | Q1: During the last month, have you woken too early and not been able to get back to sleep?  Q2: Have you had difficulty falling asleep in the last month? | **Both questions:**  Almost every night  Often  Now and again  Never | 4 answer categories. "Sometimes" & "often" = Yes. Yes on both 1) early wake and 2) initiating sleep = Insomnia | Missing = "no" if responded to one of the insomnia questions | Symptom | V Mental / behavioral |
| Depression  (HADS-D) (7) | *What describes you feeling the last week?*  I still enjoy the things I used to enjoy  I can laugh and see the funny side of things  I feel cheerful  I feel as if I'm slowed down  I have lost interest in my appearance  I look forward with enjoyment to things  I can enjoy a good book or radio/TV program | Not at all (0)  A little, but it doesn’t worry me (1)  Yes, but not too badly (2)  Very definitely and quite badly (3) | HADS-D ≥ 8.0 points | HADS-T, HADS-D and HADS-A corrected for missing at the HUNT data bank, only complete cases included | Symptom | V Mental / behavioral |
| Anxiety (HADS-A) (7) | *What describes you feeling the last week?*  I get a sort of frightened feeling like ‘butterflies’ in the stomach  Worrying thoughts go through my mind  I can sit at ease and feel relaxed  I get a sort of frightened feeling as if something awful is about to happen  I feel restless as if I have to be on the move  I get sudden feelings of panic  In the last two weeks have you felt nervous and restless? | Not at all (0)  A little, but it doesn’t worry me (1)  Yes, but not too badly (2)  Very definitely and quite badly (3) | HADS-A ≥ 8.0 | HADS-T, HADS-D and HADS-A corrected for missing at the HUNT data bank, only complete cases included. | Symptom | V Mental / behavioral |
| Epilepsy (8) | Do you have or have you ever had epilepsy? | No  Yes | Yes/no | Missing = "no" if answered any of the other yes/no questions in the same block [epilepsy, cancer, mental disease, other disease] | Diagnosis | VI Nervous system |
| Chronic headache (9) | 1) Have you been troubled by headaches in the last 12 months?  2) About how many days per month do you have a headache? | **Question 1**  Yes, in attacks (migraines)  Yes, other types of headaches  No  **Question 2**  Less than 7 days  7 to 14 days  More than 14 days | "Yes, other headache" AND "≥ 14 days" | Corrected for missing on initial headache question | Diagnosis | VI Nervous system |
| Migraine (9) | Have you been troubled by headaches in the last 12 months?  How long do the headaches last each time?  [If problems with headache]: How often is the headache characterized by or accompanied by:  Throbbing, thumping pain  Pain on one side of the head, always the same side  Nausea  Hypersensitivity to light and/or noise  Worsening with physical activity | **Question 1**  Yes, in attacks (migraines)  Yes, other types of headaches  No  **Question 2**  Less than 4 hours  4 hours – 3 days  More than 3 days  **Question 3-7**  Seldom or never  Now and again  Often | 3 migraine criteria:  1) Duration 0-72 hours  2) Migraine characteristics (”sometimes” or ”often” pulsating, unilateral, worsening physical activity)  3) 1/2 accompanying symptoms (”sometimes” or ”often” light/sound sensitive *or* nausea/vomiting)  OR  Self-reported migraine | Corrected for missing on initial headache question | Diagnosis | VI Nervous system |
| Vision impairment (10) | Q1: Do you suffer from any long-term illness or injury of a physical or psychological nature that impairs your functioning in your everyday life? (Long-term means at least one year.)  Q2: How would you describe your vision impairment? | Slight  Moderate  Severe | Answering "moderate" or "severe" = vision impairment. | Corrected for missing on initial question (Q1, chronic disease) | Symptom | VII Eye / adnexa |
| Hearing impairment (11) | Q1: Do you suffer from any long-term illness or injury of a physical or psychological nature that impairs your functioning in your everyday life? (Long-term means at least one year.)  Q2: How would you describe your hearing impairment? | Slight  Moderate  Severe | Answering "moderate" or "severe" = hearing impairment. | Corrected for missing on initial question (Q1, chronic disease) | Symptom | VIII Ear / mastoid |
| Myocardial infarction (12) | Do you have, or have you ever had heart attack? | No  Yes | Yes/no | Missing = "no" if answered any of the other yes/no questions in the same block [MI, angina, stroke, diabetes] | Diagnosis | IX Circulatory system |
| Angina pectoris (12) | Have you had or do you have angina pectoris (chest pain) | No  Yes | Yes/no | Missing = "no" if answered any of the other yes/no questions in the same block [MI, angina, stroke, diabetes] | Diagnosis | IX Circulatory system |
| Stroke / brain hemorrhage (12) | Have you had or do you have stroke/brain hemorrhage | No  Yes | Yes/no | Missing = "no" if answered any of the other yes/no questions in the same block [MI, angina, stroke, diabetes] | Diagnosis | IX Circulatory system |
| Hypertension | Mean systolic pressure measurement 2 and 3  Mean diastolic blood pressure measurement 2 and 3 |  | Blood pressure 180 > 110 *or* antihypertensive medication *without* CVD-disease | Missing CVD *OR* blood pressure medication = missing | Risk factor | IX Circulatory system |
| Asthma (13) | Do you have or have you had asthma? | No  Yes | Yes/no | No correction for asthma | Diagnosis | X Respiratory system |
| GERD (14) | To what degree have you had the following problems in the last 12 months?  Heartburn/acid regurgitation | Not at all  Slightly  Very much | "A lot heartburn/acid regurgitation last 12 months" = GERD. "No" AND "some" = not GERD. | "No" on any of the other questions in the same box [nausea, cardialgia, diarrhea, obstipation, dyspnea, tachycardia] = "no" (not missing) | Diagnosis | XI Digestive system |
| Arthritis (15) | Has a doctor ever said that you have / have had any of these diseases: arthritis (rheumatoid arthritis)? | No  Yes | Yes/no | Missing = "no" if answered any of the other yes/no questions in the same block [osteoporosis, fibromyalgia, rheumatoid arthritis, osteoarthrosis, Bechterew's disease, other musculoskeletal disease] | Diagnosis | XIII Musculoskeletal / connective tissue |
| Bechterew's disease (15) | Has a doctor ever said that you have / have had any of these diseases: Bechterew's disease (AS)? | No  Yes | Yes/no | Missing = "no" if answered any of the other yes/no questions in the same block [osteoporosis, fibromyalgia, rheumatoid arthritis, osteoarthrosis, Bechterew's disease, other musculoskeletal disease] | Diagnosis | XIII Musculoskeletal / connective tissue |
| Osteoporosis (16) | Has a doctor ever said that you have / have had any of these diseases: osteoporosis? | No  Yes | Yes/no | Missing = "no" if answered any of the other yes/no questions in the same block [osteoporosis, fibromyalgia, rheumatoid arthritis, osteoarthrosis, Bechterew's disease, other musculoskeletal disease] | Diagnosis | XIII Musculoskeletal / connective tissue |
| Fibromyalgia | Has a doctor ever said that you have / have had any of these diseases: fibromyalgia (fibrositis/chronic pain syndrome)? | No  Yes | Yes/no | Missing = "no" if answered any of the other yes/no questions in the same block [osteoporosis, fibromyalgia, rheumatoid arthritis, osteoarthrosis, Bechterew's disease, other musculoskeletal disease] | Diagnosis | XIII Musculoskeletal / connective tissue |
| Degenerative joint disease | Has a doctor ever said that you have / have had any of these diseases: degenerative joint disease (osteoarthritis)? | No  Yes | Yes/no | Missing = "no" if answered any of the other yes/no questions in the same block [osteoporosis, fibromyalgia, rheumatoid arthritis, osteoarthrosis, Bechterew's disease, other musculoskeletal disease] | Diagnosis | XIII Musculoskeletal / connective tissue |
| Chronic widespread pain (17) | During the last year, have you had pain and/or stiffness in your muscles and limbs that has lasted for at least 3 consecutive months? | No  Yes | Pain in at least one location in all three body parts: upper limb AND lower limb AND trunk | Missing = "no" if answered initial question about musculoskeletal pain | Symptom | XIII Musculoskeletal / connective tissue |
| Chronic localized pain (17) – total  10 variables | During the last year, have you had pain and/or stiffness in your muscles and limbs that has lasted for at least 3 consecutive months? | No  Yes | Neck, upper back, lower back, chest, shoulder, elbow, hand, hip, knee, ankle & NOT chronic widespread pain (total 10 variables) | Missing = "no" if answered initial question about musculoskeletal pain | Symptom | XIII Musculoskeletal / connective tissue |
| Neck pain | [If pain and/or stiffness in your muscles and limbs] Where did you have these ailments? Neck pain and/or stiffness >3months | No  Yes | Pain in the neck, but not chronic widespread pain | Missing = "no" if answered initial question about musculoskeletal pain | Symptom | XIII Musculoskeletal / connective tissue |
| Shoulder pain | [If pain and/or stiffness in your muscles and limbs] Where did you have these ailments? Shoulder pain and/or stiffness >3months | No  Yes | Pain in the shoulder but not chronic widespread pain | Missing = "no" if answered initial question about musculoskeletal pain | Symptom | XIII Musculoskeletal / connective tissue |
| Elbow pain | [If pain and/or stiffness in your muscles and limbs] Where did you have these ailments?  Elbow pain and/or stiffness >3months | No  Yes | Pain in the elbow, but not chronic widespread pain | Missing = "no" if answered initial question about musculoskeletal pain | Symptom | XIII Musculoskeletal / connective tissue |
| Wrist pain | [If pain and/or stiffness in your muscles and limbs] Where did you have these ailments?  Wrist, hand pain and/or stiffness >3months | No  Yes | Pain in the wrist, but not chronic widespread pain | Missing = "no" if answered initial question about musculoskeletal pain | Symptom | XIII Musculoskeletal / connective tissue |
| Chest pain | [If pain and/or stiffness in your muscles and limbs] Where did you have these ailments?  Chest/stomach pain and/or stiffness >3months | No  Yes | Pain in the chest, but not chronic widespread pain | Missing = "no" if answered initial question about musculoskeletal pain | Symptom | XIII Musculoskeletal / connective tissue |
| Upper back pain | [If pain and/or stiffness in your muscles and limbs] Where did you have these ailments?  Upper back pain and/or stiffness >3months | No  Yes | Pain in the upper back, but not chronic widespread pain | Missing = "no" if answered initial question about musculoskeletal pain | Symptom | XIII Musculoskeletal / connective tissue |
| Lower back pain | [If pain and/or stiffness in your muscles and limbs] Where did you have these ailments?  Lumbar region pain and/or stiffness >3months | No  Yes | Pain in the lower back, but not chronic widespread pain | Missing = "no" if answered initial question about musculoskeletal pain | Symptom | XIII Musculoskeletal / connective tissue |
| Hip pain | [If pain and/or stiffness in your muscles and limbs] Where did you have these ailments?  Hip pain and/or stiffness >3months | No  Yes | Pain in the hip, but not chronic widespread pain | Missing = "no" if answered initial question about musculoskeletal pain | Symptom | XIII Musculoskeletal / connective tissue |
| Knee pain | [If pain and/or stiffness in your muscles and limbs] Where did you have these ailments?  Knee pain and/or stiffness >3months | No  Yes | Pain in the knee, but not chronic widespread pain | Missing = "no" if answered initial question about musculoskeletal pain | Symptom | XIII Musculoskeletal / connective tissue |
| Ankle pain | [If pain and/or stiffness in your muscles and limbs] Where did you have these ailments?  Ankle, feet pain and/or stiffness >3months | No  Yes | Pain in the ankles or feet, but not chronic widespread pain | Missing = "no" if answered initial question about musculoskeletal pain | Symptom | XIII Musculoskeletal / connective tissue |
| International Prostate Symptom Score (IPSS) (18) | During the last month, how often have you had the feeling that your bladder is not completely empty after you have finished urinating?  During the last month, how often have you had to urinate again less than 2 hours after urinating?  During the last month, how often have you had to stop and start several times when urinating?  During the last month, how often has been difficult to hold back when you felt the need to urinate?  During the last month, how often have you had a weak urine flow?  During the last month, how often have you had to push or press to start urinating?  During the last month, how many times do you usually get up during the night to urinate? | **Question 1-6**  Never  About 1 of 5 times  About 1 of 3 times  About every other time  About 2 of 3 times  Almost always  **Question 7**  None  Once  2 times  3 times  4 times  5 times or more | Cut-off ≥ 7 points | Missing = "no" if answered any of the 7 IPSS questions | Symptom | XIV Genitourinary system |
| Urine incontinence (19) | Q1: Do you have involuntary loss of urine at least twice a month?  Q2: If involuntary loss of urine, how often do you have involuntary loss of urine?  Q3: If involuntary losses of urine, how much urine do you leak each time? | **Question 1**  No  Yes  **Question 2**  Less than once a month  One or more times a month  One or more times a week  Every day and/or night  **Question 3**  Drops or less  Small amount or quite a lot | Sandvik 3-level Index (volume * frequency). 6-8 points = case. | Missing = ”no” if answered both frequency and volume AND type of incontinence (stress/urge) | Symptom | XIV Genitourinary system |

### References

1. Navarro C, Chirlaque MD, Tormo MJ, Perez-Flores D, Rodriguez-Barranco M, Sanchez-Villegas A, et al. Validity of self reported diagnoses of cancer in a major Spanish prospective cohort study. J Epidemiol Community Health. 2006;60(7):593-9.

2. Brix TH, Kyvik KO, Hegedus L. Validity of self-reported hyperthyroidism and hypothyroidism: Comparison of self-reported questionnaire data with medical record review. Thyroid. 2001;11(8):769-73.

3. Midthjell K, Holmen J, Bjorndal A, Lund-Larsen G. Is questionnaire information valid in the study of a chronic disease such as diabetes? The Nord-Trondelag diabetes study. J Epidemiol Community Health. 1992;46(5):537-42.

4. Bray G, Perreault L. Obesity in adults: Prevalence, screening, and evaluation [Internet]. <www.uptodate.com> UpToDate; 2017 [updated 11.10.19; cited 12.12.2017]. Available from: <https://www.uptodate.com/contents/obesity-in-adults-prevalence-screening-and-evaluation?source=search_result&search=obesity&selectedTitle=4~150#H1387299383>

5. Selmer R, Lindman AS, Tverdal A, Pedersen JI, Njolstad I, Veierod MB. [Model for estimation of cardiovascular risk in Norway]. Tidsskr Nor Laegeforen. 2008;128(3):286-90.

6. Bonnet MH, Arand DL. Evaluation and diagnosis of insomnia in adults [Internet]. <www.uptodate.com>: UpToDate; 2019 [updated August 2019; cited 02.12.2019]. Available from: <https://www.uptodate.com/contents/clinical-features-and-diagnosis-of-insomnia-in-adults?source=search_result&search=insomnia&selectedTitle=4~150>

7. Bjelland I, Dahl AA, Haug TT, Neckelmann D. The validity of the Hospital Anxiety and Depression Scale - An updated literature review. J Psychosom Res. 2002;52(2):69-77.

8. Keezer MR, Bouma HK, Wolfson C. The diagnostic accuracy of screening questionnaires for the identification of adults with epilepsy: A systematic review. Epilepsia. 2014;55(11):1772-80.

9. Hagen K, Zwart JA, Vatten L, Stovner LJ, Bovim G. Head-HUNT: validity and reliability of a headache questionnaire in a large population-based study in Norway. Cephalalgia. 2000;20(4):244-51.

10. El-Gasim M, Munoz B, West SK, Scott AW. Discrepancies in the Concordance of Self-Reported Vision Status and Visual Acuity in the Salisbury Eye Evaluation Study. Ophthalmology. 2012;119(1):106-11.

11. Valete-Rosalino CM, Rozenfeld S. Auditory screening in the elderly: comparison between self-report and audiometry. Braz J Otorhinolaryngol. 2005;71(2):193-200.

12. Okura Y, Urban LH, Mahoney DW, Jacobsen SJ, Rodeheffer RJ. Agreement between self-report questionnaires and medical record data was substantial for diabetes, hypertension, myocardial infarction and stroke but not for heart failure. J Clin Epidemiol. 2004;57(10):1096-103.

13. Weakley J, Webber MP, Ye F, Zeig-Owens R, Cohen HW, Hall CB, et al. Agreement between obstructive airways disease diagnoses from self-report questionnaires and medical records. Prev Med. 2013;57(1):38-42.

14. Ness-Jensen E, Lindam A, Lagergren J, Hveem K. Changes in prevalence, incidence and spontaneous loss of gastro-oesophageal reflux symptoms: a prospective population-based cohort study, the HUNT study. Gut. 2012;61(10):1390-7.

15. Videm V, Thomas R, Brown MA, Hoff M. Self-reported Diagnosis of Rheumatoid Arthritis or Ankylosing Spondylitis Has Low Accuracy: Data from the Nord-Trondelag Health Study. J Rheumatol. 2017;44(8):1134-41.

16. Cunningham TD, DeShields SC. Factors associated with the accuracy of self-reported osteoporosis in the community. Rheumatol Int. 2016;36(12):1633-40.

17. Mundal I, Grawe RW, Bjorngaard JH, Linaker OM, Fors EA. Prevalence and long-term predictors of persistent chronic widespread pain in the general population in an 11-year prospective study: the HUNT study. BMC Musculoskelet Disord. 2014;15:213.

18. Barry MJ, Fowler FJ, Jr., O'Leary MP, Bruskewitz RC, Holtgrewe HL, Mebust WK, et al. The American Urological Association symptom index for benign prostatic hyperplasia. The Measurement Committee of the American Urological Association. J Urol. 1992;148(5):1549-57; discussion 64.

19. Sandvik H, Seim A, Vanvik A, Hunskaar S. A severity index for epidemiological surveys of female urinary incontinence: Comparison with 48-hour pad-weighing tests. Neurourol Urodyn. 2000;19(2):137-45.
