# Supplementary material for: Associations between complex multimorbidity, activities of daily living and mortality among older Norwegians. A prospective cohort study: the HUNT Study, Norway
Source: BMC Geriatr. 2020 Jan 21;20:21. doi: 10.1186/s12877-020-1425-3 (PMC6974981; doi:10.1186/s12877-020-1425-3)
Supplement: Supplementary file 2 — Additional file 2. Question texts, answer categories and operationalization of confounders (HUNT2). [file 12877_2020_1425_MOESM2_ESM.docx]

| **Variable (HUNT2)** | **Question text** | **Answer categories** | **Operationalization** | **Missing** |
| --- | --- | --- | --- | --- |
| Education | National register | Number of years of education | Primary education (up to 10 years)  Secondary education (10-13 years)  Tertiary education (higher education, more than 13 years) | Not applicable |
| Age | Age at participation at screening | Age in whole years | 60-69 years in HUNT2 | Not applicable |
| Gender | Gender | Man  Woman | Man  Woman | Not applicable |

**Additional File 2.** Question texts, answer categories and operationalization of confounders in HUNT2.
