# Supplementary material for: Associations between complex multimorbidity, activities of daily living and mortality among older Norwegians. A prospective cohort study: the HUNT Study, Norway
Source: BMC Geriatr. 2020 Jan 21;20:21. doi: 10.1186/s12877-020-1425-3 (PMC6974981; doi:10.1186/s12877-020-1425-3)
Supplement: Supplementary file 3 — Additional file 3. Question texts, answer categories and operationalization of outcome variables in HUNT3. [file 12877_2020_1425_MOESM3_ESM.docx]

**Additional File 3.** Question texts, answer categories and operationalization of outcome variables in HUNT3.

| **Variable (HUNT3)** | **Question text** | **Answer categories** | **Operationalization** | **Missing** |
| --- | --- | --- | --- | --- |
| Basic activities of daily living (ADL) | *Can you manage, without the help of others, in everyday life:*  Walk around indoors on the same floor  Go to the toilet  Wash yourself  Take a bath or shower  Dress and undress yourself  Go to bed and get up  Eat | No  Yes | Sum of all “no”’s  ADL dependence = ≥ 1 no | Missing if answered none of the ADL questions |
| Instrumental activities of daily living (IADL) | *Can you manage, without the help of others, in everyday life:*  Prepare warm meals  Do light housework (ex: wash dishes)  Do heavier housework (ex: wash floors)  Do heavier housework (ex: wash floors)  Do the shopping  Pay bills  Take the medicines  Go out  Take the bus | No  Yes | Sum of all no’s  IADL dependence = ≥ 1 no | Missing if answered none of the IADL questions |
| Mortality during follow-up from HUNT2 to HUNT3 | Register data | Living in the county  Moved out of the county  Deceased  Date for event | Participated in HUNT2 AND deceased before the start of HUNT3 (October 2006) | Not applicable |
| Non-participation in HUNT3 | Participated measurements, questionnaire 1 and questionnaire 2 HUNT2 | Yes, participated  Invited, but not participated | Invited, but not participated = non-participation HUNT3 | Not applicable |
