# Supplementary material for: Associations between complex multimorbidity, activities of daily living and mortality among older Norwegians. A prospective cohort study: the HUNT Study, Norway
Source: BMC Geriatr. 2020 Jan 21;20:21. doi: 10.1186/s12877-020-1425-3 (PMC6974981; doi:10.1186/s12877-020-1425-3)
Supplement: Supplementary file 4 — Additional file 4. Exposures and confounders in HUNT2 (1995–97) by outcomes in HUNT3 (2006–08). [file 12877_2020_1425_MOESM4_ESM.docx]

| **Additional File 4.** Exposures and confounders in HUNT2 (1995-97) by outcomes in HUNT3 (2006-08). | | | | | | | | | |
| --- | --- | --- | --- | --- | --- | --- | --- | --- | --- |
|  |  | No ADL (%) | Any ADL  (%) | Missing ADL (%) | No IADL  (%) | Any IADL (%) | Missing IADL (%) | Non-participation HUNT3 (%) | Mortality (%) |
| Complex multimorbidity | | |  |  |  |  |  |  |  |
|  | No | 2338 (49.4) | 49 (1.0) | 366 (7.7) | 2043 (43.2) | 333 (7.0) | 377 (8.0) | 1277 (27.0) | 701 (14.8) |
|  | Yes | 1932 (44.7) | 55 (1.3) | 310 (7.2) | 1445 (33.4) | 536 (12.4) | 316 (7.3) | 1256 (29.0) | 774 (17.9) |
|  | Total | 4270 (47.1) | 104 (1.2) | 676 (7.5) | 3488 (38.5) | 869 (9.6) | 693 (7.7) | 2533 (28.0) | 1475 (16.3) |
|  | Missing | 0 | 0 | 0 | 0 | 0 | 0 | 0 | 0 |
| Education | |  |  |  |  |  |  |  |  |
|  | Primary | 1781 (40.0) | 58 (1.3) | 327 (7.3) | 1420 (31.9) | 409 (9.2) | 337 (7.6) | 1483 (33.3) | 809 (18.2) |
|  | Secondary | 2007 (52.6) | 37 (1.0) | 299 (7.8) | 1642 (43.0) | 396 (10.4) | 305 (8.0) | 895 (23.4) | 581 (15.2) |
|  | Tertiary | 474 (62.7) | 9 (1.2) | 50 (6.6) | 419 (55.4) | 63 (8.3) | 51 (6.8) | 143 (18.9) | 80 (10.6) |
|  | Total | 4262 (47.0) | 104 (1.0) | 676 (7.0) | 3481 (38.5) | 868 (9.6) | 693 (7.7) | 2521 (28.0) | 1470 (16.0) |
|  | Missing | 8 (32.0) | 0 | 0 | 7 (28.0) | 1 (4.0) | 0 | 12 (48.0) | 5 (20.0) |
| Sex | |  |  |  |  |  |  |  |  |
|  | Women | 2340 (49.7) | 46 (1.0) | 352 (7.5) | 1971 (41.8) | 407 (8.6) | 360 (7.6) | 1393 (29.6) | 582 (12.4) |
|  | Men | 1930 (44.4) | 58 (1.3) | 324 (7.5) | 1517 (34.9) | 462 (10.6) | 333 (7.7) | 1140 (26.2) | 893 (20.6) |
|  | Total | 4270 (47.1) | 104 (1.2) | 676 (7.5) | 3488 (38.5) | 869 (9.6) | 693 (7.7) | 2533 (28.0) | 1475 (16.3) |
|  | Missing | 0 | 0 | 0 | 0 | 0 | 0 | 0 | 0 |
| Age (years) | |  |  |  |  |  |  |  |  |
|  | 60-64 | 2432 (55.4) | 45 (1.0) | 352 (8.0) | 2082 (47.5) | 387 (8.8) | 360 (8.2) | 1063 (24.2) | 495 (11.3) |
|  | 65-69 | 1838 (39.4) | 59 (1.3) | 324 (6.9) | 1406 (30.1) | 482 (10.3) | 333 (7.1) | 1470 (31.5) | 980 (21.0) |
|  | Total | 4270 (47.1) | 104 (1.2) | 676 (7.5) | 3488 (35.5) | 869 (9.6) | 693 (7.7) | 2533 (28.0) | 1475 (16.3) |
|  | Missing | 0 | 0 | 0 | 0 | 0 | 0 | 0 | 0 |
| Abbreviations used in the table: ADL = Activities of Daily Living, HUNT = the Nord-Trøndelag Health Study, IADL = Instrumental Activities of Daily Living | | | | | | | | | |
