# Supplementary material for: Associations between complex multimorbidity, activities of daily living and mortality among older Norwegians. A prospective cohort study: the HUNT Study, Norway
Source: BMC Geriatr. 2020 Jan 21;20:21. doi: 10.1186/s12877-020-1425-3 (PMC6974981; doi:10.1186/s12877-020-1425-3)
Supplement: Supplementary file 5 — Additional file 5 Association between complex multimorbidity (HUNT2) and ADL (HUNT3), mortality and non-participation (HUNT3), multinomial logistic regression.* n = 8357. [file 12877_2020_1425_MOESM5_ESM.docx]

| **Additional File 5.** Association between complex multimorbidity (HUNT2) and ADL (HUNT3), mortality and non-participation (HUNT3), multinomial logistic regression.* n=8357 | | | | | | | | | | | | | |
| --- | --- | --- | --- | --- | --- | --- | --- | --- | --- | --- | --- | --- | --- |
|  |  | ADL independent | | | ADL disability | | | Mortality during follow-up | | | Non-participation HUNT3 | | |
| CMM | | n | RR (95% CI) | RD (95% CI) | n | RR (95% CI) | RD (95% CI) | n | RR (95% CI) | RD (95% CI) | n | RR (95% CI) | RD (95% CI) |
|  | No | 2335 | 1.0 (ref) | 0.0 (ref) | 49 | 1.0 (ref) | 0.0 (ref) | 698 | 1.0 (ref) | 0.0 (ref) | 1271 | 1.0 (ref) | 0.0 (ref) |
|  | Yes | 1927 | 0.92 (0.88-0.96) | -4.3 (-6.4- -2.2) | 55 | 1.24 (0.84-1.83) | 0.3 (-0.2-0.8) | 772 | 1.23 (1.12-1.35) | 3.7 (2.0-5.2) | 1250 | 1.01 (0.95-1.08) | 0.4 (-1.6-2.4) |
|  | Total | 4262 |  | | 104 |  | | 1470 |  | | 2521 |  | |
| *Adjusted for sex, age and education.  Abbreviations used in the table: ADL = activities of daily living, CI = confidence interval, CMM = complex multimorbidity, HUNT = the Nord-Trøndelag Health Study, ref = reference category, RD = risk difference, RR = risk ratio | | | | | | | | | | | | | |
