# Supplementary material for: Associations between complex multimorbidity, activities of daily living and mortality among older Norwegians. A prospective cohort study: the HUNT Study, Norway
Source: BMC Geriatr. 2020 Jan 21;20:21. doi: 10.1186/s12877-020-1425-3 (PMC6974981; doi:10.1186/s12877-020-1425-3)
Supplement: Supplementary file 6 — Additional file 6 Association between complex multimorbidity (HUNT2) and IADL (HUNT3), mortality and non-participation (HUNT3), multinomial logistic regression.* n = 8340. [file 12877_2020_1425_MOESM6_ESM.docx]

| **Additional File 6.** Association between complex multimorbidity (HUNT2) and IADL (HUNT3), mortality and non-participation (HUNT3), multinomial logistic regression.* n=8340 | | | | | | | | | | | | | |
| --- | --- | --- | --- | --- | --- | --- | --- | --- | --- | --- | --- | --- | --- |
|  |  | IADL independent | | | IADL disability | | | Mortality during follow-up | | | Non-participation HUNT3 | | |
| CMM | | n | RR (95% CI) | RD (95% CI) | n | RR (95% CI) | RD (95% CI) | n | RR (95% CI) | RD (95% CI) | n | RR (95% CI) | RD (95% CI) |
|  | No | 2040 | 1.0 (ref) | 0.0 (ref) | 333 | 1.0 (ref) | 0.0 (ref) | 698 | 1.0 (ref) | 0.0 (ref) | 1271 | 1.0 (ref) | 0.0 (ref) |
|  | Yes | 1441 | 0.78 (0.75-0.83) | -10.0 (-12.1- -8.0) | 535 | 1.80 (1.58-2.05) | 6.0 (4.7-7.4) | 772 | 1.23 (1.12-1.34) | 3.6 (2.0-5.2) | 1250 | 1.01 (0.95-1.08) | 0.4 (-1.6-2.4) |
|  | Total | 3481 |  | | 868 |  |  | 1470 |  |  | 2521 |  |  |
| *Adjusted for age, sex and education.  Abbreviations used in the table: CI = confidence interval, CMM = complex multimorbidity, HUNT = the Nord-Trøndelag Health Study, IADL = instrumental activities of daily living, ref = reference category, RD = risk difference, RR = risk ratio | | | | | | | | | | | | | |
